# Variable-intercept panel model for deformation zoning of a super-high arch dam

**DOI:** 10.1186/s40064-016-2600-z

**Published:** 2016-06-27

**Authors:** Zhongwen Shi, Chongshi Gu, Dong Qin

**Affiliations:** State Key Laboratory of Hydrology-Water Resources and Hydraulic Engineering, Hohai University, Nanjing, 210098 China; National Engineering Research Center of Water Resources Efficient Utilization and Engineering Safety, Hohai University, Nanjing, 210098 China; College of Water-Conservancy and Hydropower Engineering, Hohai University, Nanjing, 210098 China

**Keywords:** Deformation zoning, Variable-intercept panel model, Super-high arch dam, Similarity index, Validity checking

## Abstract

This study determines dam deformation similarity indexes based on an analysis of deformation zoning features and panel data clustering theory, with comprehensive consideration to the actual deformation law of super-high arch dams and the spatial–temporal features of dam deformation. Measurement methods of these indexes are studied. Based on the established deformation similarity criteria, the principle used to determine the number of dam deformation zones is constructed through entropy weight method. This study proposes the deformation zoning method for super-high arch dams and the implementation steps, analyzes the effect of special influencing factors of different dam zones on the deformation, introduces dummy variables that represent the special effect of dam deformation, and establishes a variable-intercept panel model for deformation zoning of super-high arch dams. Based on different patterns of the special effect in the variable-intercept panel model, two panel analysis models were established to monitor fixed and random effects of dam deformation. Hausman test method of model selection and model effectiveness assessment method are discussed. Finally, the effectiveness of established models is verified through a case study.

## Background

The entire service period of a super-high arch dam can be divided into several stages, and each stage presents a different deformation behavior pattern. Although plenty of analysis models have been developed for the complex influencing factors and deformation law of super-high arch dams, the majority of these models are just extension of the traditional safety monitoring model of dam deformation. Analysis models are based on one-dimensional time series of single measuring point. For this reason, the effects of measurement error, missing data, and collinearity on model precision are difficult to avoid. Moreover, spatial–temporal monitoring information on super-high arch dam deformation cannot simply be randomly obtained. The spatial–temporal deformation features of the entire dam structure throughout its service period, as well as their correlations, are difficult to comprehend. Therefore, the traditional deformation analysis model fails to completely reflect the deformation behavior of a super-high arch dam. In addition, influencing factors (e.g., load effect, constraint, material property, and environmental factors) of the deformation behavior differ significantly at different regions of a super-high arch dam. However, the traditional method does not consider such difference and still analyzes all measuring points with reservoir water level, temperature, and aging. The actual deformation laws of different regions of a super-high arch dam are difficult to depict. Therefore, an analysis model that can reflect the different influencing factors of deformation should be established.

Panel data contain deformation information of two dimensions—time and cross section—which adequately reflect the spatial–temporal features of arc deformation. A deformation panel sequence is superior to pure time series and cross section sequence for rich information, high degree of freedom (DOF), and effective collinearity reduction. Hence, panel data can be used for modeling an analysis of the deformation behavior of a super-high arch dam. Before establishing the panel model, all monitoring points on the super-high arch dam were zoned according to deformation similarity with consideration for regional characteristics of overall deformation and correlation of deformation at all measuring points, which can eliminate the influence of different deformation laws at different monitoring points and measuring error on the model. Furthermore, because common influencing factors (reservoir water level, temperature and aging) cannot easily depict the different deformation laws of different regions, this study introduced dummy variables that can represent the effect of special influencing factors of different regions, called special deformation effect variables. On this basis, an analysis model that considers both common and special influencing factors of deformation is established, which has higher explanatory power and estimation precision. Meanwhile, studying special deformation effect variables is beneficial in comprehensively analyzing the deformation behavior of the super-high arch dams and is expected to offset some shortcomings of the traditional analysis model.

## Criteria for dam deformation zoning

Dam deformation zoning analysis has to address two key problems: (1) determining the statistical magnitude that can be used to represent the deformation similarity between measuring points and (2) determining the specific systematic zoning method to be used, that is, the criteria to be used to determine deformation similarity between different dam regions. The criteria that would determine the deformation similarity of different dam regions were constructed based on the panel features of super-high arch dam deformation combined with spatial–temporal information of deformation. The criteria created the deformation zoning method for the super-high arch dam by studying structural deformation behavior in time dimension and cross-section dimension.

### Deformation similarity indexes

To solve the first key problem, this study constructed deformation similarity indexes based on the idea of panel data clustering.

**Fig. 1:**
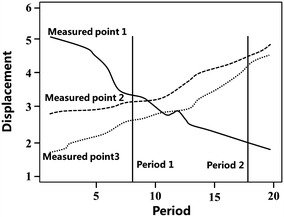
Monitoring value of measured points during different periods

The deformation zoning of a super-high arch dam makes the deformation law within the same region as similar as possible and makes the deformation laws of different regions as different as possible; such consideration is an important standard in measure the dam deformation zoning effect. Traditional deformation zoning applies the mean time series of deformation of all measuring points. In other words, it degenerates deformation sequence into a cross-section sequence. This method can express only the mean variation of dam deformation and loses the temporal information of deformation. In addition, it makes an invisible assumption that temporal deformation at different measuring points changes toward the same direction, which cannot easily reflect the variation law of deformation behavior with time. This assumption is unreasonable. As shown in Fig. 1, measuring points 1 and 3 shall be divided into the same type in a traditional deformation zoning. However, the variation of deformation sequence throughout the service period is considered, measuring points 2 and 3 shall be divided into the same type.

The deformation data of super-high arch dams reveal at least three aspects: (1) the absolute deformation of the arch dam; (2) the dynamic level of time series of deformation, that is, the deformation growth with time; and (3) the fluctuation of deformation development, that is, the degree of variation or fluctuation. The focus of this chapter is dam deformation zoning based on the similarity of deformation sequence when the dam structure has not obviously changed or the time series of the deformation that has smoothly change. The similarity of the deformation sequence is reflected by combining the “absolute deformation” and “deformation growth” of the dam.

#### Measuring method of indexes

**Fig. 2:**
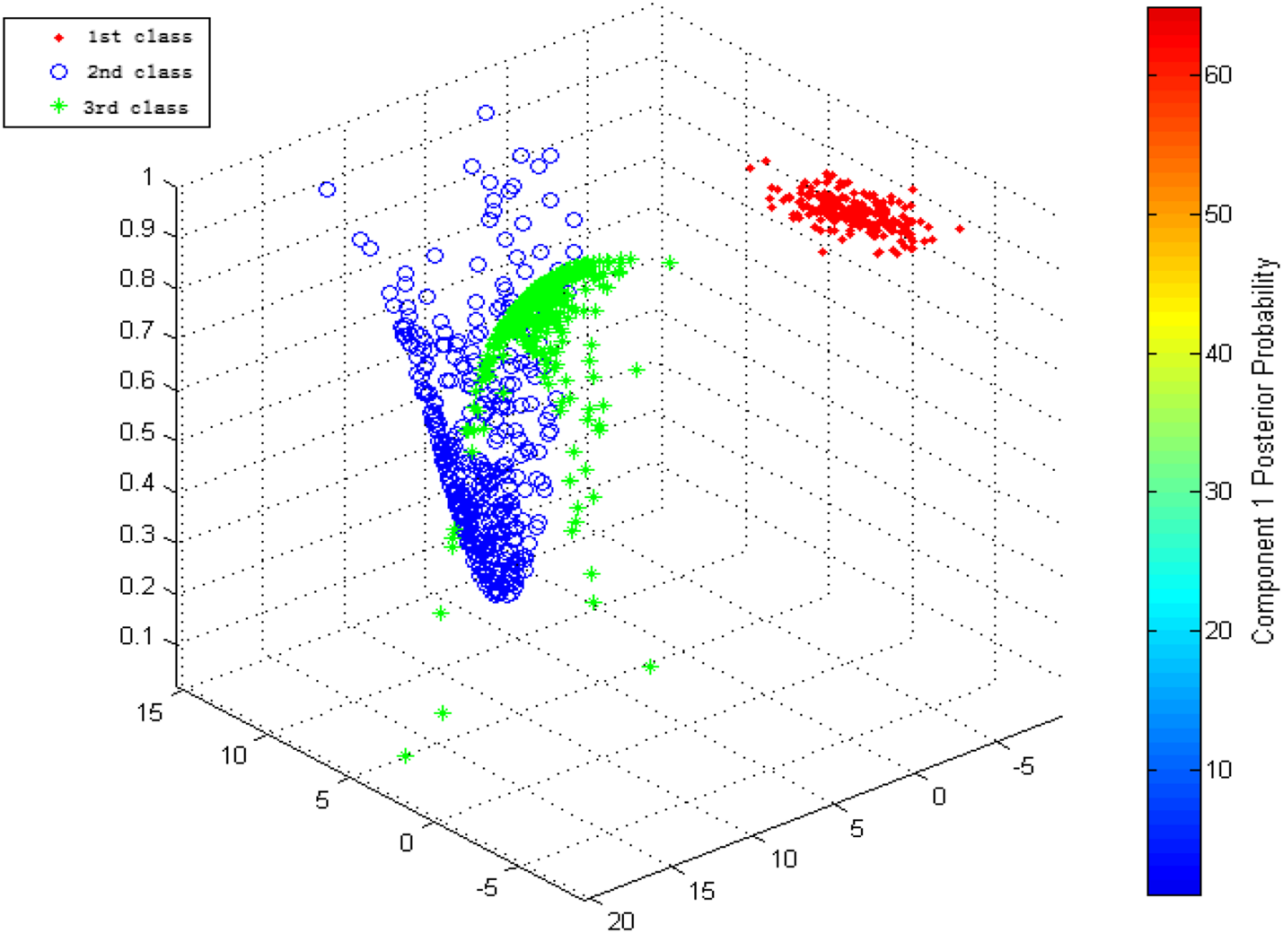
Clustering sketch map based on distance similarity

When analyzing deformation monitoring results, “absolute deformation” and “deformation growth” can be depicted by some distances (Fig. 2). Common distance functions include Euclidean distance, absolute distance (Block distance), Chebyshev distance, Minkowski distance, and Mahalanobis distance (Li and He 2010). With the comprehensive difference of absolute value and dynamic development trend of arc development taken into consideration, the deformation similarity between different measuring points was described by Euclidean distance in this study. A distance measurement formula for spatial deformation similarity indexes was given in Euclidean distance.

During deformation data preprocessing, *δ*_*it*_(*i* = 1, 2, …, *N*; *t* = 1, 2, …, *T*) is often used to express the deformation dataset of the super-high arch dam, where *N* is the total measuring points on the dam, and *T* is the total monitoring periods, that is, monitoring time series. With respect to the deformation dataset, *δ*_*it*_, *S*_*t*_ is defined as the standard deviation (SD) of deformation during *t*, and *d*_*ij*_ is the direct distance between measuring points *i* and *j*. *d*_*ij*_ meets the following basic axiom (He 2008).

##### **Definition 1**

The “absolute” distance between measuring points *i* and *j* is recorded as *d*_*ij*_(*AQED*):

1 $$d_{ij} = (AQED) = \left[ {\sum\limits_{t = 1}^{T} {(\delta_{it} } - \delta_{jt} )^{2} } \right]^{1/2}$$

where *δ*_*it*_ is the deformation of measuring point *i* during *t*, *δ*_*jt*_ is the deformation of measuring point *j* during *t*, and *d*_*ij*_(*AQED*) is the distance between measuring points *i* and *j* during the entire *T*.

##### **Definition 2**

The “growing” distance between measuring points *i* and *j* is recorded as *d*_*ij*_(*ISED*):

2 $$d_{ij} (ISED) = \left[ {\sum\limits_{t = 1}^{T} {\left( {\frac{{\Delta \delta_{it} }}{{\Delta \delta_{it - 1} }} - \frac{{\Delta \delta_{jt} }}{{\Delta \delta_{jt - 1} }}} \right)}^{2} } \right]^{1/2}$$

where Δ*δ*_*it*_ = *δ*_*it*_ − *δ*_*it*−1_ and Δ*δ*_*jt*_ = *δ*_*jt*_ − *δ*_*it*−1_. Δ*δ*_*it*_ and Δ*δ*_*jt*_ are the difference of the absolute deformation between two adjacent periods. *d*_*ij*_(*ISED*) describes the difference of the deformation growth between measuring points *i* and *j* with time. If deformations of measuring points *i* and *j* change toward the same direction, the higher harmony of such change will lead to a higher deformation similarity between these two measuring points and smaller *d*_*ij*_(*ISED*). If deformations of measuring points *i* and *j* change toward the opposite directions, the change will cause a poorer deformation similarity and a larger *d*_*ij*_(*ISED*). These conditions conform to the basic principle of similarity measurement.

To accurately describe the deformation characteristics of different measuring points, establishing a comprehensive criterion that can depict deformation similarity is necessary. As a result, the “comprehensive” distance between measuring points *i* and *j* is introduced, which is recorded as *d*_*ij*_(*CED*):

3 $$d_{ij} (CED) = \omega_{1} d_{ij} (AQED) + \omega_{2} d_{ij} (ISED)$$

where *ω*_1_ and *ω*_2_ represent weights of two distances, which meet *ω*_1_ + *ω*_2_ = 1.

*d*_*ij*_(*CED*) is the weighted array of the “absolute” distance and the “growing” distance. The weight coefficient can be determined subjectively or objectively according to practical situations of the problem being studied. To comprehensively reflect the total information of spatial deformation data of the super-high arch dam, the weight coefficient that conforms to practical engineering significance was given. In this study, the weight coefficient of the “comprehensive” distance was calculated through an entropy weight method. Based on the idea of information entropy and comprehensive considerations to the evaluation system of distance indexes, the method assumed that *m* evaluation objects and *n* evaluation indexes exist. Then, the original data can be expressed by matrix *R* = (*r*_*ij*_)_m*n_ as

4 $$R = \left( {\begin{array}{*{20}c} {r_{11} } & {r_{12} } & \cdots & {r_{1m} } \\ {r_{21} } & {r_{22} } & \cdots & {r_{2m} } \\ \vdots & \vdots & \vdots & \vdots \\ {r_{n1} } & {r_{n2} } & \cdots & {r_{nm} } \\ \end{array} } \right)_{n*m}$$

where *r*_*ij*_ is the evaluation value of the *j*th evaluation object under the *i*th evaluation index. Combined with abovementioned deformation similarity indexes of ultrahigh arc dam, the “absolute” distance and the “growing” distance were used as the evaluation indexes of deformation, and the distance between two measuring points is viewed as the evaluation object.

1. Calculate the characteristic proportion of the *i*th evaluation index to the *j*th evaluation object as
  5 $$p_{ij} = \frac{{r_{ij} }}{{\sum\nolimits_{j = 1}^{m} {r_{ij} } }},\quad (i = 1,2, \ldots n;j = 1,2, \ldots m)$$
  where *p*_*ij*_ ϵ [0, 1] and the original proportional relationship between deformation monitoring sequences remains undamaged, that is, $d_{ij} \ge 0,\,\,\,\sum\nolimits_{i = 1}^{N} {d_{ij} > 0}$.
2. Calculate the entropy. The entropy of the *i*th evaluation index values is given as
  6 $$S_{i} = - \frac{1}{\ln m}\sum\limits_{j = 1}^{m} {p_{ij} } \ln p_{ij} ,\quad (i = 1,2, \ldots n).$$
3. Determine the entropy weight. The entropy weight of the *i*th evaluation index is
  7 $$w_{i} = \frac{{1 - S_{i} }}{{\sum\nolimits_{i = 1}^{n} {1 - S_{i} } }},\quad (i = 1,2, \ldots n).$$

The statistical magnitude of the similarity measurement is defined by Euclidean distance. The calculated *w*_*i*_ is the weight coefficient of the *i*th evaluation index. Substituting into Eq. (3) obtains *d*_*ij*_(*CED*) between the different measuring points can be used as the criterion of the deformation similarity. In this way, the first key problem, that is, determining the statistical magnitude that can be used to represent deformation similarity between measuring points, is solved. Similar statistical magnitudes can be acquired according to other common distance forms (e.g., Mahalanobis distance and Lance distance) or correlation coefficient and included angle cosine. These other distance forms, coefficient, and angle cosine are not introduced in this paper (Li and He 2010). In the following text, the number of dam zones with different deformation similarities is determined through panel data system clustering, thus proposing the systematic dam deformation zoning method.

#### Determining the number of dam deformation zones and zoning process

To address the second key problem, this study assumes that *N* measuring points of the dam are divided into *k* regions () based on Ward clustering and combined with the proposed similarity measurement *G*_1_, *G*_2_, …, *G*_*k*_. Let *N*_*l*_ represent the number of *G*_*l*_ measuring points, $\overline{{X_{l} }}$ be the mean measured value of *G*_*l*_ measuring points, and *X*_*il*_ be the deformation of the *i*th measuring point (*i* = 1, 2, …, *N*_*l*_) in *G*_*l*_. For the deformation data of *N* measuring points during *T*, the sum of squares of deviations of sequence at different measuring points in *G*_*l*_ is

8 $$W_{l}^{*} = \sum\limits_{i = 1}^{{N_{l} }} {\sum\limits_{t = 1}^{T} {\left[ {\omega_{1} (X_{it} - \overline{{X_{t} }} )^{\prime } (X_{it} - \overline{{X_{t} }} )} \right]} } + \sum\limits_{t = 2}^{T} {\left[ {\omega_{2} (Y_{it} - \overline{{Y_{t} }} )'(Y_{it} - \overline{{Y_{t} }} )} \right]} .$$

The total sum of squares of deviations of *k* regions is

9 $$W^{*} = \sum\limits_{l = 1}^{k} {W_{l}^{*} } .$$

In Eq. (8), *W*_*l*_ is the total sum of squares of the deviations of *N*_*l*_ measured values, *X*_*it*_ is the deformation of measuring point *i* during *t*, $$Y_{it} = \frac{{\Delta X_{it} }}{{X_{it - 1} }}$$ is the relative deformation growth of measuring point *i* in *G*_*l*_ during *t*, Δ*X*_*it*_ = *X*_*it*_ − *X*_*it*−1_ is the difference of absolute deformation of measuring point *i* in *G*_*l*_ between *t* and $$t - 1$$$$\overline{{X_{t} }} = \frac{1}{{N_{l} }}\sum\nolimits_{t = 1}^{{N_{l} }} {X_{it} }$$, and $$\overline{{Y_{t} }} = \frac{1}{{N_{l} }}\sum\nolimits_{t = 1}^{{N_{l} }} {Y_{it} }$$.

According to the above clustering method, given fixed *k*, the deformation zoning with minimum *W* is the optimum. In practical engineering, because of the complexity of the super-high arch dam structural system, predetermining *k* is actually an artificial interference to dam deformation zoning, which is likely to cause subjective errors. However, increase in *k* will cause continuous changes of similarity distance in the same region and between different regions. Therefore, obtaining absolute optimum *k* is impossible. A method that determines the threshold of dam deformation zoning is proposed in this paper based on a thresholding method (Li and He 2010; He 2008; Kaitai and Enpei 1982; Huixuan 2005). A total of *n* combinations are assumed to be implemented during the zoning process. The regional distance ratio between the *l*th zoning and the last zoning (*S*_*l*_) is calculated as

10 $$S_{l} = \frac{{D_{l} }}{{D_{n - 1} }}.$$

If *S*_*l*_ differs slightly with *S*_*l*+1_, but significantly differs with *S*_*l*−1_, corresponding regional distance *D*_*l*_ can be used as the threshold of deformation zoning. Based on this threshold, the number of regions can be further calculated.

The implementation method and steps to the second key problem are interpreted based on Ward clustering. A complete dam deformation zoning process is proposed in next according to deformation similarity criteria and determination method of *k*.

Suppose super-high arch dam has *N* deformation measuring points during *T*. On the basis of the basic idea of the proposed dam deformation zoning, the deformation distance between different measuring points and distance between deformed dam regions are calculated first. During the initialization, these *N* deformation monitoring points are classified into the same class. The regional distance and the distance between deformation sequences of different measuring points are equal. Next, two measuring points with the closest distance are combined into a new region, of which distance with other regions will be calculated again. Later, the dam is rezoned according to the principle of minimum sum of squares of deviations (*W).* The process is repeated until all measuring points are combined into one region. The specific steps are as follows:

- Step 1 Calculate the “absolute” and “growing” deformation distances and regional distances through Eqs. (1) and (2).
- Step 2 Calculate the entropy weights above two distances through Eqs. (5), (6), and (7).
- Step 3 Substitute the calculated weight coefficients into Eq. (3) to calculate comprehensive distance *d*_*ij*_(*CED*) between two measuring points and matrix of regional distance *D*^(0)^.
- Step 4 Initialize (Step 1, *i* = 1) all measuring points into one region, that is, *k* = *N*. Let *D*^(1)^ = *D*^(0)^ and the *i*th region be *G*_*i*_ = {*X*_(*i*)_}(*i* = 1, 2, …, *N*).
- Step 5 Calculate regional distance matrix *D*^(*i*−1)^, and combine two regions with the minimum “comprehensive distance” into a new region according to the principle of minimum *W*.
- Step 6 Calculate comprehensive distance (_*dij*_(*CED*)) between the new region with other regions and obtain new distance matrix *D*^*i*^. Repeat Steps 5 and 6 until all measuring points are divided into one region.
- Step 7 Draw the hierarchical dendrogram.
- Step 8 Derive the optimum zoning scheme and the optimum number of zoning (*K*) according to method to determine the dam deformation zoning threshold and practical situations. Draw the dam deformation zoning distribution map.

**Fig. 3:**
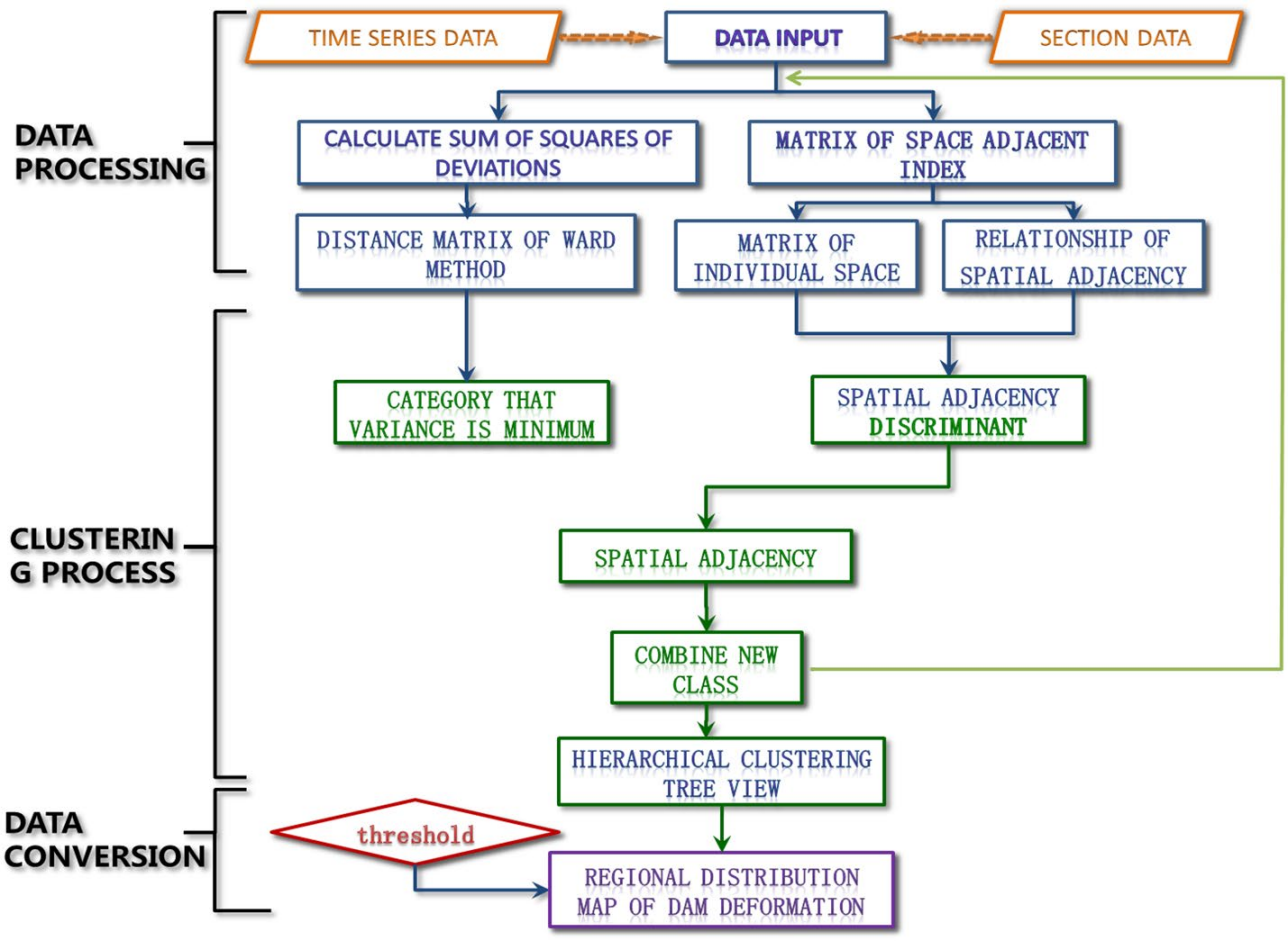
Flowchart of the partitions of the dam deformation

The flowchart of the deformation zoning for the super-high arch dam is shown in Fig. 3.

## Establishment of variable-intercept panel model for deformation

### Selection of deformation components

**Fig. 4:**
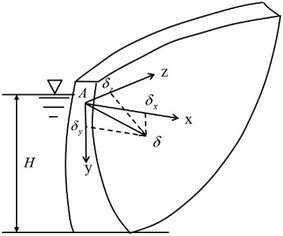
Sketch map of the deformation vector and its component

Except for hydraulic pressure (water level) and temperature changes, aging is another influencing factor of super-high arch dam deformation. In recent years, P Lin, among others, studied how deformation is influenced by arch dam seepage, crack, etc. (Lin et al. 2013, 2014a, b). Deformation at any point of the arc dam can be viewed as a deformation vector (*δ*) that can be decomposed into radial horizontal deformation (*δ*_*x*_), tangential horizontal deformation (*δ*_*y*_), and vertical deformation (*δ*_*z*_) (Fig. 4). Every vector component is divided into hydraulic pressure component (*δ*_*H*_), temperature component (*δ*_*T*_), and aging component (*δ*_*θ*_):11

**Fig. 5:**
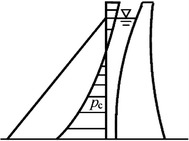
Load distribution of the cantilever bean of the arch dam

For the ultrahigh arc dam, the hydraulic pressure load distributed on the girder (*P*_*c*_) makes nonlinear changes because of the combined action of the horizontal arc and cantilever beam (Fig. 5). Hence, *P*_*c*_ is generally expressed by the quadratic or cubic expressions of upstream depth of water (*H*):

12 $$P_{c} = \sum\limits_{i = 1}^{2(3)} {a_{i}^{\prime } H^{i} } .$$

*P*_*c*_*H* Deformations caused by *P*_*c*_ (*δ*_1*H*_ and *δ*_2*H*_) are related with *H*, *H*^2^, *H*^3^, and *H*^4^ (or *H*^5^) because and have quadratic or cubic curve relations. *δ*_3*H*_ is similar with *δ*_1*H*_ and *δ*_2*H*_. Therefore, hydraulic pressure component of the super-high arch dam can be expressed as

13 $$\delta_{H} = \sum\limits_{i = 1}^{4(5)} {a_{i} } H^{i} .$$

Temperature component *δ*_*T*_ refers to the deformation caused by temperature changes of concrete and basement of the arc dam. In terms of mechanics, *δ*_*T*_ shall choose temperatures of dam concrete and basement reading by the thermometer as factors. With respect to arch dam under normal operation, when hydration heat of concrete has been dissipated and a quasi-equilibrium temperature field is developed inside the dam body, dam temperature is determined by boundary temperature changes. At this moment, the periodic term combined by multiple harmonic waves can be used as the factor of *δ*_*T*_:

14 $$\delta_{T} = \sum\limits_{i = 1}^{2} {\left( {b_{1i} \sin \frac{2\pi it}{365} + b_{2i} \cos \frac{2\pi it}{365}} \right)} .$$

**Fig. 6:**
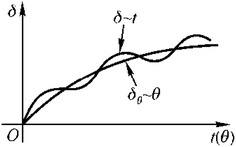
Changing law of time-dependent deformation ()

Aging-induced dam deformation (*δ*_*θ*_) has complex causes. It comprehensively reflects the creep of dam concrete, the creep deformation of dam basement, and compressive deformation of the basement tectonics. The variation law of *δ*_*θ*_ of common dams under normal operation is shown in Fig. 6.

**Fig. 7:**
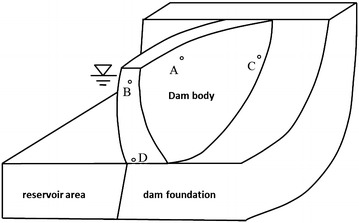
Sketch map of the specific effect quantities at different measuring points of the high arch dam

Various function forms are options in establishing the mathematical model of *δ*_*θ*_. With the deformation characteristics of super-high arch dam taken into consideration, the mathematical model is expressed by a logarithmic function in this study as

15 $$\delta_{\theta } = c_{1} \theta + c_{2} \ln \theta$$

where *H*, *T*, and *θ* are hydraulic pressure, temperature, and aging, respectively. In practical engineering (Fig. 7), most deformations at monitoring points *A, B, C,* and *D*, which are are caused by applied load, can be interpreted by common influencing factors (hydraulic pressure, temperature, and aging). However, the deformation at *C* and *D*, which are close to the dam foundation and bank slope, differs significantly from that at *A* and *B*, which are close to the dam top. This finding is closely related with the synergistic effect of constraints, material properties, and surrounding environments at different dam regions. As a result, different parts produce different special deformation effects (*α*). Parameter heterogeneity of the deformation analysis model will occur once the explained variables fail to capture these complex factors that can not be monitored and quantized. Although the traditional deformation analysis model can depict the main influencing factors of the deformation using the independent variables of the model, the model often neglects deformation specificity at different measuring points caused by these complex factors. In particular, different deformation effects at different measuring points have to be taken into account for a super-high arch dam structure with large span. Based on the panel data theory, *α*_*i*_ that is used to represent special deformation effect is introduced to establish a variable-intercept panel model for deformation zoning of the super-high arch dam.

### Variable-intercept panel model for dam deformation zoning

#### Expression form of the variable-intercept panel model

The general form of variable-intercept panel model is

16 $$Y_{it} = {\varvec{\upbeta}}X_{it} + \alpha + u_{it} \quad \left( {i = 1,2,3 \ldots N;\,t = 1,2,3 \ldots T} \right)$$

where *Y*_*it*_ is the deformation panel sequence of *i* measuring points; $$Y_{it} = \left[ {\begin{array}{*{20}c} {y_{11} } & {y_{12} } & \cdots & {y_{1t} } \\ {y_{21} } & {y_{22} } & \cdots & {y_{2t} } \\ \vdots & \vdots & \ddots & \vdots \\ {y_{i1} } & {y_{i2} } & \cdots & {y_{it} } \\ \end{array} } \right]$$ (*i* = 1,2, … *N*; t = 1,2, … *T*), *y*_*it*_ is the monitored deformation of the *i*th measuring points during *t*, *X*_*it*_ is an independent variable (*x*_*it*_ is a 1 × *K* vector, and *K* is number of independent variables), and $$X_{it} = \left[ {\begin{array}{*{20}c} {x_{11} } & {x_{12} } & \cdots & {x_{1t} } \\ {x_{21} } & {x_{22} } & \cdots & {x_{2t} } \\ \vdots & \vdots & \ddots & \vdots \\ {x_{i1} } & {x_{i2} } & \cdots & {x_{it} } \\ \end{array} } \right]$$. The main influencing factors of deformation were chosen as the calculation formula, combined with mechanical properties of ultrahigh arc dam. Then, $x_{it} = (1,\,H_{t}^{1} ,\,H_{t}^{2} ,\,H_{t}^{3} ,\,H_{t}^{4} ,\,T_{1,t} ,\, \ldots ,\,T_{m,t} ,\,\theta_{t} ,\,\ln \theta_{t} )^{\prime }$. *α* is a scalar constant that represents the special deformation effect at different dam regions caused by special influencing factors. Explicitly including these special influencing factors (e.g., dam structure, constraint, material properties, and load) into independent variables of the model is difficult. Therefore, *α* is employed to absorb individual special deformation effects caused by these factors. $\beta = (a_{0} ,\,a_{1} ,\,a_{2} ,\,a_{3} ,\,a_{4} ,\,b_{1} ,\, \ldots ,\,b_{m} ,\,c_{1} ,\,c_{2} )^{\prime }$ is the parameter that has to be estimated. *u*_*it*_ is an independent identically distributed random error component with a mean value of 0 and a variance of σ^2^.

#### Panel model of fixed deformation effect

On the basis of the general expression of the panel model [Eq. (16)], *α*_*i*_ that varies at different measuring points is introduced into the model. Then, the panel model of fixed deformation effect of super-high arch dam is

17 $$\delta = F\left( \bullet \right) + \alpha_{i} + \varepsilon = f\left( {H,T,\theta } \right) + \alpha_{i} + \varepsilon$$

where $F\left( \bullet \right)$ is a continuous regression the function that meets certain conditions and serves as the explanatory variable in the panel model of fixed deformation effect, *H* is the depth of water, *T* is the thermometer read value, *θ* is an influencing factor that is related with time, *α*_*i*_ is the fixed deformation effect at different measuring points, and *ε* is the random error term.

The panel model of fixed deformation effect can be expressed as a matrix, that is,

18 $$Y_{it} = X_{it} {\varvec{\upbeta}} + \alpha_{i} + u_{it} ,\quad (i = 1,2,3 \cdots N;\quad t = 1,2,3 \cdots T)$$

where *Y*_*it*_ represents all the monitoring sequences of *i* measuring points, *X*_*it*_ is an independent variable that reflects the main influencing factors of deformation, parameter **β** is independent from measuring points *i* and time *t*, ${\varvec{\upbeta}} = (a_{0} ,\,a_{1} ,\,a_{2} ,\,a_{3} ,\,a_{4} ,\,b_{1} ,\, \ldots ,\,b_{m} ,\,c_{1} ,\,c_{2} )^{\prime }$, and *α*_*i*_ is the unique fixed deformation effect of different measuring points, which depicts the special deformation effect at different dam positions and deformation differences between different dam positions. At this moment, $\alpha_{i} = (\alpha_{1} ,\alpha_{2} ,\alpha_{3} \ldots \alpha_{N} )$ and **β** are two fixed parameters that have to be estimated. Then, the panel model matrix of fixed deformation of the super-high arch dam can be expressed as

19 $$Y = \left[ {\begin{array}{*{20}c} {y_{1} } \\ \vdots \\ {y_{i} } \\ {y_{N} } \\ \end{array} } \right] = \left[ {\begin{array}{*{20}c} e \\ 0 \\ \vdots \\ 0 \\ \end{array} } \right]\alpha_{1} + \left[ {\begin{array}{*{20}c} 0 \\ e \\ \vdots \\ 0 \\ \end{array} } \right]\alpha_{2} + \cdots + \left[ {\begin{array}{*{20}c} 0 \\ 0 \\ \vdots \\ e \\ \end{array} } \right]\alpha_{N} + \left[ {\begin{array}{*{20}c} {x_{1} } \\ \vdots \\ {x_{i} } \\ {x_{N} } \\ \end{array} } \right]\beta + \left[ {\begin{array}{*{20}c} {u_{1} } \\ \vdots \\ {u_{i} } \\ {u_{N} } \\ \end{array} } \right],$$

where

20 $$y_{i} = \left[ {\begin{array}{*{20}c} {y_{i1} } \\ {y_{i2} } \\ \vdots \\ {y_{iT} } \\ \end{array} } \right],\quad x_{i} = \left[ {\begin{array}{*{20}c} {x_{1it} } & {x_{2it} } & \cdots & {x_{Kit} } \\ {x_{1i2} } & {x_{2i2} } & \cdots & {x_{Ki2} } \\ \vdots & \vdots & {} & \vdots \\ {x_{1iT} } & {x_{2iT} } & \cdots & {x_{KiT} } \\ \end{array} } \right],\quad e = \left[ {\begin{array}{*{20}c} 1 \\ 1 \\ \vdots \\ 1 \\ \end{array} } \right],\quad {\text{and}}\quad u_{i} = \left[ {\begin{array}{*{20}c} {u_{i1} } \\ {u_{i2} } \\ \vdots \\ {u_{iT} } \\ \end{array} } \right].$$

#### Panel model of random deformation effect

If the special deformation effect of different measuring points is viewed as a random variable and the random effect model is used to describe the actual state of dam deformation so that the model parameters focus on the main components of monitored deformation, the random effect will reflect the special deformation component of different measuring points. In other words, individual effect in the variable-intercept panel model ($\eta_{i}$) is viewed as a random variable. The following text studies the panel model of monitored random deformation effect.

According to Eqs. (16) and (17), the panel model of monitored random deformation effect can be expressed in the following matrix:

21 $$Y_{it} = X_{it} {\varvec{\upbeta}} + \alpha_{i} + \varepsilon_{it} ,\quad (i = 1,2,3 \cdots N;\quad t = 1,2,3 \cdots T),$$

where *Y*_*it*_ represents all monitoring sequences of the dam; *X*_*it*_ is an independent variable, $$X_{it} = \left[ {\begin{array}{*{20}c} {x_{11} } & {x_{12} } & \cdots & {x_{1t} } \\ {x_{21} } & {x_{22} } & \cdots & {x_{2t} } \\ \vdots & \vdots & \ddots & \vdots \\ {x_{i1} } & {x_{i2} } & \cdots & {x_{it} } \\ \end{array} } \right]$$, $x_{it} = (1,\,H_{t}^{1} ,\,H_{t}^{2} ,\,H_{t}^{3} ,\,H_{t}^{4} ,\,T_{1,t} ,\, \ldots ,\,T_{m,t} ,\,\theta_{it} ,\,\ln \theta_{it} )^{\prime }$; $H_{t}^{1}$, $H_{t}^{2}$, $H_{t}^{3}$, and $H_{t}^{4}$ are the influencing factors of *δ*_*H*_; *T*_1,*t*_, …, *T*_*m*,*t*_ are influencing factors of *δ*_*T*_; and *θ*_*it*_ and ln*θ*_*it*_ are the influencing factors of *δ*_*θ*_. ${\varvec{\upbeta}} = (a_{0} ,\,a_{1} ,\,a_{2} ,\,a_{3} ,\,a_{4} ,\,b_{1} ,\, \ldots ,\,b_{m} ,\,c_{1} ,\,c_{2} )^{\prime }$. *ε*_*it*_ meets $E(\left. {\varepsilon_{it} } \right|x_{i1} , \cdots ,x_{iT} ) = 0$, and $\varepsilon_{it} \mathop \sim\limits^{iid} (0,\sigma_{\varepsilon }^{2} )$; *α*_*i*_ is random deformation effect of different measuring points. For *i*, *j*, and *t*, *α*_*i*_ meets $E(\left. {\alpha_{it} } \right|x_{i1} , \ldots ,x_{iT} ) = 0$. $E(\alpha_{i}^{2} ) = \sigma_{\alpha }^{2}$, $E(\alpha_{i} \alpha_{j} ) = 0,i \ne j$, and $E(\varepsilon_{it} \alpha_{j} ) = 0$ represent special effect of external complex factors on deformation at different dam positions. Special effect of every measuring point is a random variable. Specificity of the overall dam deformation conforms to normal distribution. Deformation difference between different dam positions can be further reflected by distribution of *α*_*i*_.

In summary, two kinds of influencing factors of super-high arch dam deformation (*Y*) exist: independent variables and special effect. The independent variables $X_{1} , \ldots ,X_{p}$ represent common influencing factors of deformation at all measuring points (hydraulic pressure, temperature, and aging), whereas special effect $\alpha_{1} , \ldots ,\alpha_{i}$ reflects deformation difference between different measuring points. Such special effect has two values, and the corresponding panel model has a fixed-effect model and random-effect model. In practical engineering, the panel model that conforms to deformation characteristics of the dam shall be chosen, which means that choosing the appropriate model according to the test results of the deformation monitoring sequences is necessary.

#### Model selection and effectiveness evaluation

Deformation monitoring sequences of the super-high arch dam can be used to establish the zoning panel model by using the proposed method. However, dummy variables that reflect deformation difference between different measuring points, that is, whether the special effect is a fixed effect or a random effect, shall be determined by the specification test of the panel model. In the panel model, the OLS estimator that gained by meeting the basic regression hypothesis is a BLUE estimator. If $E(v_{it} \left| {X_{it} ) = 0} \right.$ is not met, the generalized least square estimator $\hat{\beta }_{GLS}$ is inconsistent. Therefore, fixed-effect and random-effect models shall be differentiated. Choosing the fixed-effect or random-effect model can be decided according to a correlation test between random error term and variables (Lin et al. 2013, 2014a, b; Hausman 1978), that is, true or false test of $E(v_{it} \left| {X_{it} ) = 0} \right.$. Hausman assumed that

22 $$\hat{q}_{1} = \hat{\beta }_{GLS} - \hat{\beta }_{within}$$

where $\hat{\beta }_{within}$ is a within estimator. The null hypothesis is $H_{0} :E(v_{it} \left| {X_{it} ) = 0} \right.$, and the alternative hypothesis is $H_{1} :E(v_{it} \left| {X_{it} ) \ne 0} \right.$.

On this basis, two statistical variables can be added into the test to ensure the applicability of the deformation panel model. Set

23 $$\hat{q}_{2} = \hat{\beta }_{GLS} - \hat{\beta }_{Between} \quad {\text{and}}\quad \hat{q}_{3} = \hat{\beta }_{within} - \hat{\beta }_{Between}$$

where $\hat{\beta }_{Between}$ is a between estimator. Then, the test statistics obtained are

24 $$m_{2} = \hat{q}^{\prime}_{2} [\text{var} (\hat{q}_{2} )]^{ - 1} \hat{q}_{2} \quad {\text{and}}\quad m_{3} = \hat{q}^{\prime}_{3} [\text{var} (\hat{q}_{3} )]^{ - 1} \hat{q}_{3} .$$

In the null hypothesis, when $H_{0} :E(v_{it} \left| {X_{it} ) = 0} \right.$ is true, the asymptotic distributions of both *m*_2_ and *m*_3_ are $\chi_{K}^{2}$.

Based on the above analysis, if $E(v_{it} \left| {X_{it} ) = 0} \right.$, the factors in the model, which can not be monitored, change randomly and are uncorrelated with independent variables. Under this circumstance, the random-effect model should be chosen. If $E(v_{it} \left| {X_{it} ) = 0} \right.$ is false, the factors in the model that cannot be monitored are correlated with independent variables, and their effect on the model can be tested. Therefore, the fixed-effect model shall be chosen.

When evaluating the effect of a model, the main consideration is whether explanatory variables of the model can interpret changes of dependent variables as much as possible. The evaluation requires some reference standard or guidelines; otherwise, it cannot determine whether the chosen model is good, appropriate, or accurate in the empirical analysis (Hausman and Taylor 1981). In this study, the effect of the established variable-intercept panel model was evaluated from the overall goodness of fit and significance of every variable.

The overall goodness of fit is measured with corrected sample determination coefficient (*R*^2^) and *F*. It is defined as

25 $$R^{2} = \frac{ESS}{TSS};\quad F = \frac{{{{R^{2} } \mathord{\left/ {\vphantom {{R^{2} } {\left( {k - 1} \right)}}} \right. \kern-0pt} {\left( {k - 1} \right)}}}}{{{{\left( {1 - R^{2} } \right)} \mathord{\left/ {\vphantom {{\left( {1 - R^{2} } \right)} {\left( {n - k} \right)}}} \right. \kern-0pt} {\left( {n - k} \right)}}}}$$

where *ESS* is the regression sum of squares, *TSS* is the total sum of squares of deviations, *n* is the sample size, and *k* is the number of explanatory variables, including the intercept.

According to the definition of *R*^2^, the regression fitting effect of estimation improves as *R*^2^ approaches 1. The *R*^2^ growth in, which is influenced by increasing explanatory variables, is unrelated with the fitting effect because the panel model contains plenty of explanatory variables. *R*^2^ must be adjusted: dividing the residual sum of squares and *TSS* by their DOF to eliminate the effect of variable amount on goodness of fit. The adjusted *R*^2^ is defined as

26 $$\bar{R}^{2} = 1 - \frac{RSS/(n - k - 1)}{TSS/(n - 1)}$$

where *RSS* is the residual sum of squares, *TSS* is the total sum of squares of deviations, *n* − *k* − 1 is the DOF of *RSS*, and *n* − 1 is the DOF of *TSS*.

Moreover, the overall significance test can be implemented by using *F* statistics. The *F* test statistics is defined as

27 $$F = \frac{ESS/k}{RSS/n - k - 1} = \frac{{\sum\nolimits_{i = 1}^{n} {(\hat{y}_{i} - \bar{y})^{2} /k} }}{{\sum\nolimits_{i = 1}^{n} {(y_{i} - \hat{y})^{2} /n - k - 1} }}\sim\,F(k,n - k - 1)$$

where *ESS* is the regression sum of squares, and *RSS* is the residual sum of squares. When the null hypothesis is true, the statistics conform to the *F* distribution whose DOF is $(k,n - k - 1)$. Numerical value *P* can be gained by calculating *F*. Given significance level *α*, the truth of falsity of the null hypothesis can be determined by comparing *F*_*α*_ and *P*. According to above analysis, *F* and *R*^2^ move together. Hence, the effectiveness and goodness of fit of the model can be evaluated comprehensively through adjusted *R*^2^ and *F*. The higher the *F* and *R*^2^ (Maddala 1971; Mundlak 1978; Chen and Li 2006; Damodar 2000; Hsiao 1985), the better the fitting effect of the model.

Moreover, the significance of every variables in the panel model can be tested through *t*-statistic. *t*-statistic is defined as

28 $$t = \frac{{\hat{\beta }_{i} }}{{S_{{\hat{\beta }_{i} }} }}\sim\,t(n - k - 1) .$$

If many insignificant variables exist in the model after one *t* test, the variable with the minimum *t* shall be deleted, and then, another *t* test is performed. Only one variable can be deleted in every *t* test. The process is repeated until all variables pass through the *t* test.

## Case study

**Fig. 8:**
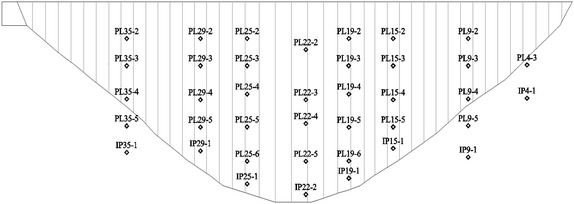
Location distribution graph of the monitoring points of the *vertical line*

Deformation of a super-high arch dam was zoned using the established zoning method and the special effect panel model. The studied super-high arch dam is a parabolic dome dam, which has a crest elevation of 1, 245 m. The dam is 294.5 m high, 901.771 m long at the dam crest, 12 m wide at the crown cantilever top, and 72.912 m wide at the bottom. It has a complex structure. To improve the stress distribution at the dam heel, a structure joint with an elevation of 956 m and a crack depth of 9 m is set at the dam heel. A total of 39,741 dam deformation monitoring data of 39 vertical line points buried in the dam are taken as the research object. All data were collected from August 9, 2009, to July 24, 2012. The distribution of vertical line points is shown in Fig. 8. The objective of this case study is to analyze and verify the effectiveness of the proposed deformation zoning method for super-high arch dam and the established variable-intercept panel model for deformation zoning.

### Deformation zoning of the super-high arch dam

**Fig. 9:**
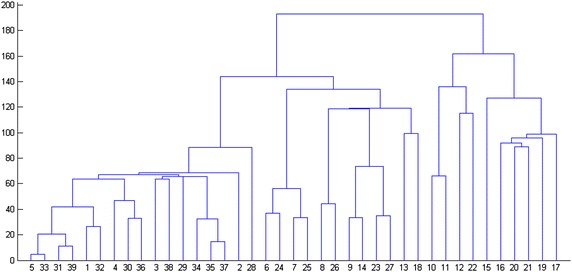
Hierarchical clustering tree view of the measure points of the dam

**Fig. 10:**
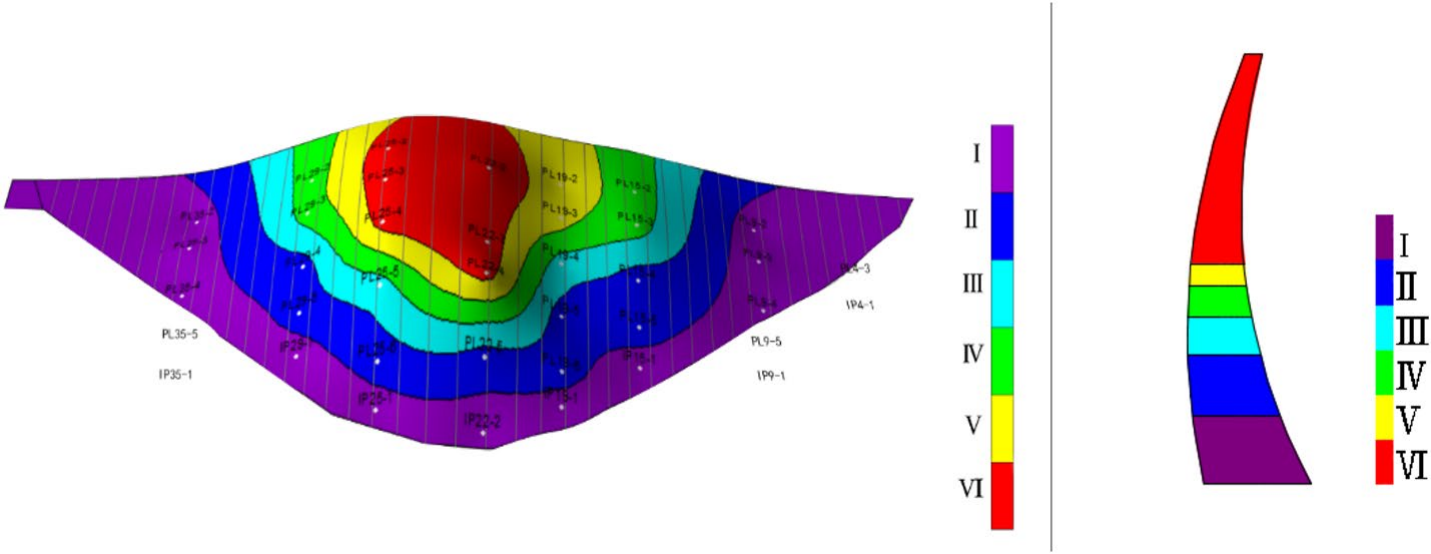
Regional distribution map of dam deformation

Individual similarity index is used to calculate Eqs. (1) and (2), which is expressed in Euclidean distance. The 39 measuring points of the dam are divided into different deformation zones according to the deformation zoning flowchart of the super-high arch dam (Fig. 3), obtaining the hierarchical dendrogram (Fig. 9). Multiple evaluations were implemented with internal effectiveness indexes, and the optimum number of dam deformation zones is determined by combining the trial-and-error iteration and the optimum zoning threshold. Finally, all measuring points are divided into six groups. The measuring points of the same cluster present the same deformation variation trend and law. Each group of measuring points can comprehensively describe the overall deformation features of the corresponding dam area. The entire super-high arch dam can be divided into six regions according to locations of measuring points (Fig. 10).

### Model selection

To choose the appropriate regression model to establish the panel model for deformation zoning of the super-high arch dam, Hausman test was implemented to deformation time series of all measuring points based on the dam deformation zoning. In other words, the appropriate panel model (fixed-effect model or random effect model) shall be chosen according to the correlation test between random error term and variables. In this study, Hausman test was implemented to the deformation time series of all six deformation zones. The test results of two typical zones are listed in Tables 1 and 2.

**Table 1:** Hausman test result of deformation time series of the measuring points in the area of section 1 of the dam

Hausman test	
b = consistent under Ho and Ha; obtained from xtreg	
B = inconsistent under Ha, efficient under Ho; obtained from xtreg	
Test: Ho: difference in coefficients not systematic	
chi2(2) = (b-B)’[(V_b-V_B)^(−1)](b-B) = 0.00	
Prob > chi2 = 1.0000

**Table 2:** Hausman test result of deformation time series of the measure points in the area of Sect. 3 of the dam

Hausman test	
b = consistent under Ho and Ha; obtained from xtreg	
B = inconsistent under Ha, efficient under Ho; obtained from xtreg	
Test: Ho: difference in coefficients not systematic	
chi2(2) = (b−B)’[(V_b−V_B)^(−1)](b−B) = 9.14	
Prob > chi2 = 0.0104	

According to the Hausman test results, Wald test statistics Prob (hereinafter referred as P), Eq. (22), and the null hypothesis $E(v_{it} \left| {X_{it} ) = 0} \right.$, P of the rest zones, except zone III, fails to turn down the null hypothesis under 5 % significance level, indicating that P conforms to the random-effect model. P of zone III is equal to 0.0104, which implies that it can turn down the null hypothesis under 5 % significance level, can use the fixed-effect model.

### Variable-intercept panel model for deformation zoning

Based on the dam deformation zoning and Hausman test results, a modeling analysis was performed using the corresponding variable-intercept panel model for deformation zoning, thus obtaining coefficients and test statistics of the influencing factors. The results of zones I and III are shown in Tables 3 and 4.

**Table 3:** Results of the random-effects model of the panel data in the area of section I of the dam

Random-effects model						
Random-effects GLS regression			Number of obs = 17323			
Group variable: _j			Number of groups = 17			
R-sq: within = 0.0000			Obs per group: min = 1019			
between = 0.0000			avg = 1019.0			
overall = 0.1816			max = 1019			
			Wald chi2(10) = 15548.80			
corr(u_i, X) = 0 (assumed)			Prob > chi2 = 0.0000			

var	Coef.	Std.Err.	z	P > |z|	[95 % Conf. Interval]	
x1	−159.307	827.6679	−0.19	0.847	−1781.51	1462.893
x2	331.9333	1494.828	0.22	0.824	−2597.88	3261.743
x3	−280.182	1192.674	−0.23	0.814	−2617.78	2057.416
x4	91.74331	354.8331	0.26	0.796	−603.717	787.2034
x5	0.067455	0.118763	0.57	0.57	−0.16532	0.300226
x6	−0.97668	0.111943	−8.72	0	−1.19608	−0.75727
x7	−0.01332	0.042572	−0.31	0.754	−0.09676	0.070115
x8	−0.3112	0.020144	−15.45	0	−0.35068	−0.27172
x9	0.006649	0.019384	0.34	0.732	−0.03134	0.04464
x10	−0.05327	0.020773	−2.56	0.01	−0.09398	−0.01255
_cons	23.4151	170.7861	0.14	0.891	−311.32	358.1497
sigma_u	3.061757					
sigma_e	1.702147					
rho	0.763903	(fraction of variance due to u_i)

**Table 4:** Results of the fixed-effects model of the panel data in the area of section III of the dam

Fixed-effects model						
Fixed-effects (within) regression			Number of obs = 2038			
Group variable: _j			Number of groups = 2			
R-sq: within = 0.9602			Obs per group: min = 1019			
between = .			avg = 1019.0			
overall = 0.9602			max = 1019			
			F(10,2026) = 4892.37			
corr(u_i, X) = 0 (assumed)			Prob > F = 0.0000			

var	Coef.	Std.Err.	z	P > |z|	[95 % Conf. Interval]	
x1	−8441.51	2756.353	−3.06	0.002	−13847.1	−3035.93
x2	15257.36	4978.174	3.06	0.002	5494.487	25020.23
x3	−12008.4	3971.919	−3.02	0.003	−19797.8	−4218.88
x4	3522.763	1181.688	2.98	0.003	1205.313	5840.214
x5	6.01408	0.395511	15.21	0	5.238429	6.789732
x6	−2.62441	0.372799	−7.04	0	−3.35552	−1.8933
x7	−0.78513	0.141776	−5.54	0	−1.06317	−0.50709
x8	−1.45153	0.067085	−21.64	0	−1.58309	−1.31997
x9	−0.10303	0.064552	−1.6	0.111	−0.22963	0.023561
x10	−0.43057	0.06918	−6.22	0	−0.56624	−0.2949
_cons	1710.663	568.7574	3.01	0.003	595.2524	2826.073
sigma_u	0.02987277					
sigma_e	1.944306					
rho	0.000236	(fraction of variance due to u_i)				
F test that all u_i = 0: F(1, 2026) = 0.24 Prob > F = 0.6239						

**Fig. 11:**
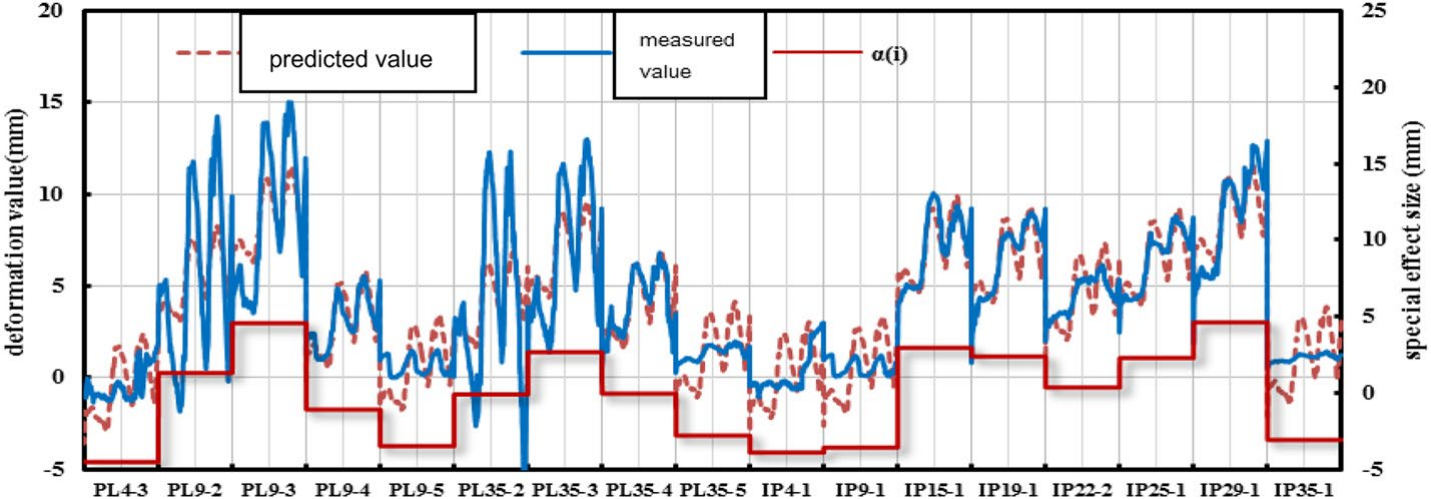
Deformation measured-fitting process line of measure points in section 1 of the dam

**Fig. 12:**
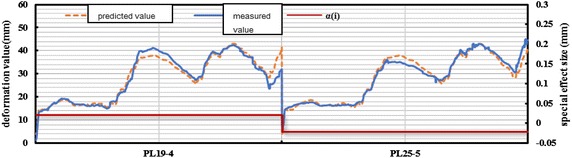
Deformation measured-fitting process line of measure points in section 3 of the dam

An appropriate panel model is established for different deformation zones according to the method for solving fixed-effect and random-effect models. Hydrographs of the measured value and the fitting value of panel models are presented in Figs. 11 and 12. A special effect (*α*_*i*_) of every measuring point is given explicitly. Deformation difference within the same zone (*α*_*i*_) conforms to the known random distribution. The analysis on six deformation panel models reveals that: (1) the model is established with full consideration to the difference of different measuring points. $prob > chi2 = 0$, which indicates the overall significance of the parameters. The parameters improve explanatory power of the model and enable to get a comprehensive understanding on deformation features of corresponding dam zones, laying foundations for further analysis of structural changes, deformation forecasting and early warning, dam evolution, etc. Through a comparative analysis of error variance (sigma_e), special effect variance (sigma_u), and proportion of special effect in the entire fluctuation (rho = sigma_u/(sigma_e + sigma_u)) of different deformation zones, the combination of explanatory variables and the introduced dummy variables are to be confirmed reasonable and can reflect dam deformation law well.

According to the comparison of the modeling results of six deformation zones, no significant individual difference is observed among measuring points of the same zone. For measuring points of the same zone, max(Δ*α*_*i*_) = 10.331 mm, which reflects that the structural deformations of six divided zones make the same responses to external complex factors and can be viewed as the structural deformation of the same nature in the analysis and computation.

## Conclusions

1. According to the regional difference of dam deformation, in this study, all measuring points of a super-high arch dam are divided into six zones on the basis of deformation similarity. Modeling analysis is implemented to every zone on the basis of the monitored deformation, thereby effectively eliminating the interference of the deformation difference among different measuring points and measurement errors in the model.
2. The effects of the special influencing factors of different dam zones on dam deformation was analyzed based on the basis of the studied deformation features of the super-high arch dam. Dummy variables that represent the special effects of dam deformation were introduced to establish the variable-intercept panel model for the deformation zoning of the super-high arch dam. The variable-intercept panel models, except those for zones I and II, can adequately explain the actual deformation behavior of the dam, as manifested by the fitting effect. Furthermore, the inherent differences in the deformation laws among different dam zones can be discovered by analyzing the special deformation effect.
3. By considering the different patterns of the special effect in the variable-intercept panel model, two panel models consistent with practical engineering situations were established based on the Hausman test: the fixed-effect model the and random-effect model. The evaluation method of model effectiveness was explored.
4. Although the random-effect model can adequately explain the overall dam deformation behavior, an accurate fitting is difficult to achieve if only the special deformation effect is involved. For example.in this study, the deformation laws in zones I and II, which are related to various factors, such as constraints and regional difference of foundation properties, are very complex because these two zones are close to the dam foundation. Therefore, dynamic adjustment of the model coefficient is needed during modeling analysis, which requires further research.

## References

[CR1] Chen X, Li G (2006) Spatial panel data model analysis of economic convergence in China. Econ Sci (5) (in Chinese)

[CR2] Damodar N (2000). Gujarati. Econometrics.

[CR3] Hausman JA (1978). Specification tests in econometrics. Econometrica.

[CR4] Hausman JA, Taylor WE (1981). Panel data and unobservable individual effects. Econometrica.

[CR5] He X (2008). Multivariate statistical analysis.

[CR6] Hsiao C (1985). Benefits and limitations of panel data. Econom Rev.

[CR7] Huixuan G (2005). Application of multivariate statistical analysis.

[CR8] Kaitai F, Enpei P (1982). Cluster analysis.

[CR9] Li Y, He X (2010). Panel clustering method and its application. Stat Res.

[CR10] Lin P, Liu XL, Hu Y, Xu WB, Li QB (2013). Deformation stability analysis of Xiluodu arch dam under stress-seepage coupling condition. Chin J Rock Mech Eng.

[CR11] Lin P, Zhou WY, Liu HY (2014). Experimental study on cracking, reinforcement, and overall stability of the Xiaowan super-high arch dam. Rock Mech Rock Eng.

[CR12] Lin P, Ma TH, Liang ZZ, Tang CA, Wang RK (2014). Failure and overall stability analysis on high arch dam based on DFPA code. Eng Fail Anal.

[CR13] Maddala GS (1971). The likelihood approach to pooling cross-section and time series data. Econometrica.

[CR14] Mundlak Y (1978). On the pooling of time series and cross-section data. Econometrica.

